# Human Biosample Authentication Using the High-Throughput, Cost-Effective SNPtrace^TM^ System

**DOI:** 10.1371/journal.pone.0116218

**Published:** 2015-02-25

**Authors:** May M. Y. Liang-Chu, Mamie Yu, Peter M. Haverty, Julie Koeman, Janet Ziegle, Marie Lee, Richard Bourgon, Richard M. Neve

**Affiliations:** 1 Department of Discovery Oncology, Genentech, Inc., 1 DNA Way, South San Francisco, CA 94080, United States of America; 2 Department of Bioinformatics and Computational Biology, Genentech, Inc., 1 DNA Way, South San Francisco, CA 94080, United States of America; 3 Fluidigm Corporation, 7000 Shoreline Court, Suite 100, South San Francisco, CA 94080, United States of America; 4 Van Andel Research Institute, Cytogenetics, Grand Rapids, MI 49503, United States of America; Universitat Pompeu Fabra, SPAIN

## Abstract

Cell lines are the foundation for much of the fundamental research into the mechanisms underlying normal biologic processes and disease mechanisms. It is estimated that 15%–35% of human cell lines are misidentified or contaminated, resulting in a huge waste of resources and publication of false or misleading data. Here we evaluate a panel of 96 single-nucleotide polymorphism (SNP) assays utilizing Fluidigm microfluidics technology for authentication and sex determination of human cell lines. The SNPtrace Panel was tested on 907 human cell lines. Pairwise comparison of these data show the SNPtrace Panel discriminated among identical, related and unrelated pairs of samples with a high degree of confidence, equivalent to short tandem repeat (STR) profiling. We also compared annotated sex calls with those determined by the SNPtrace Panel, STR and Illumina SNP arrays, revealing a high number of male samples are identified as female due to loss of the Y chromosome. Finally we assessed the sensitivity of the SNPtrace Panel to detect intra-human cross-contamination, resulting in detection of as little as 2% contaminating cell population. In conclusion, this study has generated a database of SNP fingerprints for 907 cell lines used in biomedical research and provides a reliable, fast, and economic alternative to STR profiling which can be applied to any human cell line or tissue sample.

## Introduction

Human cell lines and patient-derived tissue are an essential resource for biomedical research. As the need for these resources grows in industry and academia, so have examples of biosample mix-ups [1–5]. The simple fact is that human error leads to mistakes which, unless stringent quality controls are in place, can result in costly mistakes, false and invalid data and retractions of publications [6–10]. For example, serial biopsies, matched normal and diseased tissue or samples for personal genomics are often acquired for comparative studies and the results of such studies are invalid if these tissues are mixed up [11]. Cell lines require propagation and cryopreservation which can lead to misidentification or cross-contamination, and it is estimated that between 15–35% of human cell line cultures are contaminated in some manner [10]. This has dramatic repercussions, represents a huge waste of resources and contributes to the irreproducibility of scientific data. The current standard for authenticating human samples is short tandem repeat (STR) profiling. This is a multiplexed PCR-based assay which measures tetranucleotide repeats at up to 16 loci and is an American Type Culture Collection (ATCC) standardized method also recognized by the American National Standards Institute (ANSI) [2,12,13]. Detection of single-nucleotide polymorphisms (SNPs) is another established method for authenticating biologic samples [14–21]. However, SNP profiling has not gained significant traction in our community as an alternative or complement to STR profiling. Here we evaluate the SNPtrace Panel, a new high-throughput panel of 96-SNP assays utilizing Fluidigm microfluidics technology, for authentication and sex identification of human cell lines and tissues. The SNPtrace Panel was used to profile 907 samples for which we had also generated STR profiles. We report the performance of the assay in comparison to STR profiling based on ability to discriminate related and unrelated samples, sex calls and intra-human cross-contamination and we provide a database of SNP calls for 907 individual cell lines.

## Material and Methods

### Cell Lines

907 cell lines were used in this study (**S1 Table**). These consisted of 820 genetically distinct lines. 170 samples consisted of cell lines with one or more related cell lines within the panel derived from the same patient, termed synonymous (for example CX-1, HT-29 and WiDr) or misidentified (for example HeLA, BT-B) or derivative (for example FA9JTO, FA9JTOTERT). STR and the SNPtrace Panel profiles for all lines can be found in **S1 Table**.

### The SNPtrace Panel SNPs

The SNPtrace Panel is a set of SNPtype assays designed to enable sample tracking and fingerprinting by genotyping 96 SNP loci across the human genome. The SNP loci consist of 90 autosomal loci and six allosome loci. Sex calls from the SNPtrace Panel are determined by 6 allosome SNPs (3 Chr X SNPs and 3 Chr Y SNPs), with redundancy to ensure that a sample can be sex-typed if one assay were to fail. The 90 autosomal loci include 46 loci that are polymorphic across all populations [19] and 44 ancestry-informative loci [22]. Some SNPs were also chosen to correspond with commercial arrays. A list of the SNPtrace Panel SNP identifiers and their chromosomal location can be found in **S4 Table**.

### DNA preparation and pre-amplification

DNA was extracted from frozen cell pellets ranging from 3e6 to 5e6 cells using either DNeasy Blood & Tissue (Qiagen, cat#69506) or 96 Blood & Tissue Kit (Qiagen, cat#69581) following manufacturer’s protocol. DNA concentration was determined by NanoDrop 8000 and normalized to 50ng/ μL.

All samples were pre-amplified in 96-well plates as described in the Fluidigm SNP Genotyping User Guide. Briefly, all samples were pre-amplified in 96-well plates with the 10X SNPtrace Panel specific target amplification (STA) primer pool, prepared by pooling 2 μl of all 96 pairs of 100 μM STA and 100 μM locus specific primer (LSP) primers diluted with DNA suspension buffer to a final concentration of 500 nM for each primer. In a DNA free hood, 60 μL of 10X SNPtype STA Primer Pool was combined with 300 μL of Qiagen 2X Multiplex PCR Master Mix (Qiagen, PN 206143) and 90 μL of PCR-certified water to create the STA pre-mix. Next, 3.75 μL of the STA pre-mix was combined with 1.25 μL of genomic DNA and thermal cycled with a 15 minute 95°C hot start and 14 cycles alternating between 95°C for 15 seconds and 60°C for 4 minutes (**S5 Table**). After thermal cycling, the specific target amplification reaction was diluted 100 times and was used for the SNPtrace Panel genotyping and identification as described below. Sample preparation: 10 min. Machine time: 1 h 15 min.

### The SNPtrace Panel Genotyping Reaction

The genotyping reactions were created as described in the Fluidigm SNP Genotyping User Guide. The genotyping assays were created by combining 3 μL of each allele-specific primer (ASP) pool and 8 μL of each locus-specific primer (LSP) for each of the 96 SNPtrace Panel assays into a 96 well plate with 29 μL of DNA suspension buffer. Next, 1 μL of each assay was combined with 2.5 μL of 2X Assay Loading Reagent (Fluidigm, PN 85000738) and 1.5 μL of PCR-certified water creating 5 μL of the assay mix and 4 μL of each 96 assay mixes was transferred to the assay inlets. Following this, 2.5 μL of the 1:100 diluted pre-amplification reaction (see above) was combined with 3 μL of Biotium 2X Fast Probe Master Mix (Biotium, PN 31005), 0.3 μL of 20X SNPtype sample loading reagent (Fluidigm, PN 100-3425), 0.1 μL of 60X SNPtype Reagent (Fluidigm, PN 100-3402), 0.036 μL of ROX (Invitrogen, PN 12223-012) and 0.064 μL of PCR-certified water. Finally, 5 μL of each sample was transferred to the sample inlets of the Fluidigm High Precision 96.96 Genotyping integrated fluidic circuit (IFC) for genotyping.

Once the samples and assays were transferred, the IFC was inserted into the Fluidigm IFC Controller, HX, which created 9,216 reaction chambers. The IFC was thermal cycled and end point imaged on the BioMark HD System under conditions outlined in **S5 Table**. The data from the IFC were initially analyzed on the Fluidigm SNP Genotyping Analysis Software. Sample preparation (96-samples): 30 min mixing, 10 min transferring samples to IFC. Machine time (96-samples): 20 min priming, 1 h 30 min loading, 1 h 30 min thermal cycling/reading. Data analysis ~30 sec/sample (clean run) or 10 min/sample (more complex samples).

### STR Analysis

All human cell lines used in this publication were processed by Genetica DNA Laboratories (a LabCorp Specialty Testing Group; Burlington, NC) for authentication testing using analytical procedures for DNA extraction (see above), polymerase chain reaction (PCR) and capillary electrophoresis on a 3130xl genetic analyzer (Applied Biosystems). The thirteen core CODIS short tandem repeat (STR) loci plus PENTA E and PENTA D, and the sex-specific locus, amelogenin, were analyzed using the commercially available PowerPlex 16HS amplification kit (Promega Corporation) and GeneMapper ID v3.2.1 software (Applied Biosystems). Appropriate positive and negative controls were used concurrently throughout the analysis. Sample preparation (16 samples): 30 min, 1 min (transfer). Machine run time (16 samples): DNA amplification, 2 hr; genetic analyser, 1h; Data analysis ~30 sec/sample (clean run) or 10 min/sample (more complex samples).

### Pairwise comparison identity scores

For either SNP or STR data, we followed Tanabe et al. [23] and computed an identity score for any pair of samples as follows: for each locus at which sample 1 and sample 2 both had called alleles (i.e., where neither is a “no call”), we computed (i) the total number of distinct alleles seen in sample 1, (ii) the total number of distinct alleles seen in sample 2, and (iii) the number of distinct alleles shared by both samples. Each of the three counts was then summed across all loci, and the identity score was defined as *2 x shared / (total 1 + total 2)*. The identity score is 0 if and only if no common alleles are seen at any locus; it is 1 if and only if the exact same alleles are seen in both samples at all loci. Note that this approach does not assume diploid genomes or biallelic markers, nor does it require that the same set of markers be available for every pair of samples.

### *P*-values for relatedness

**Fig 1:**
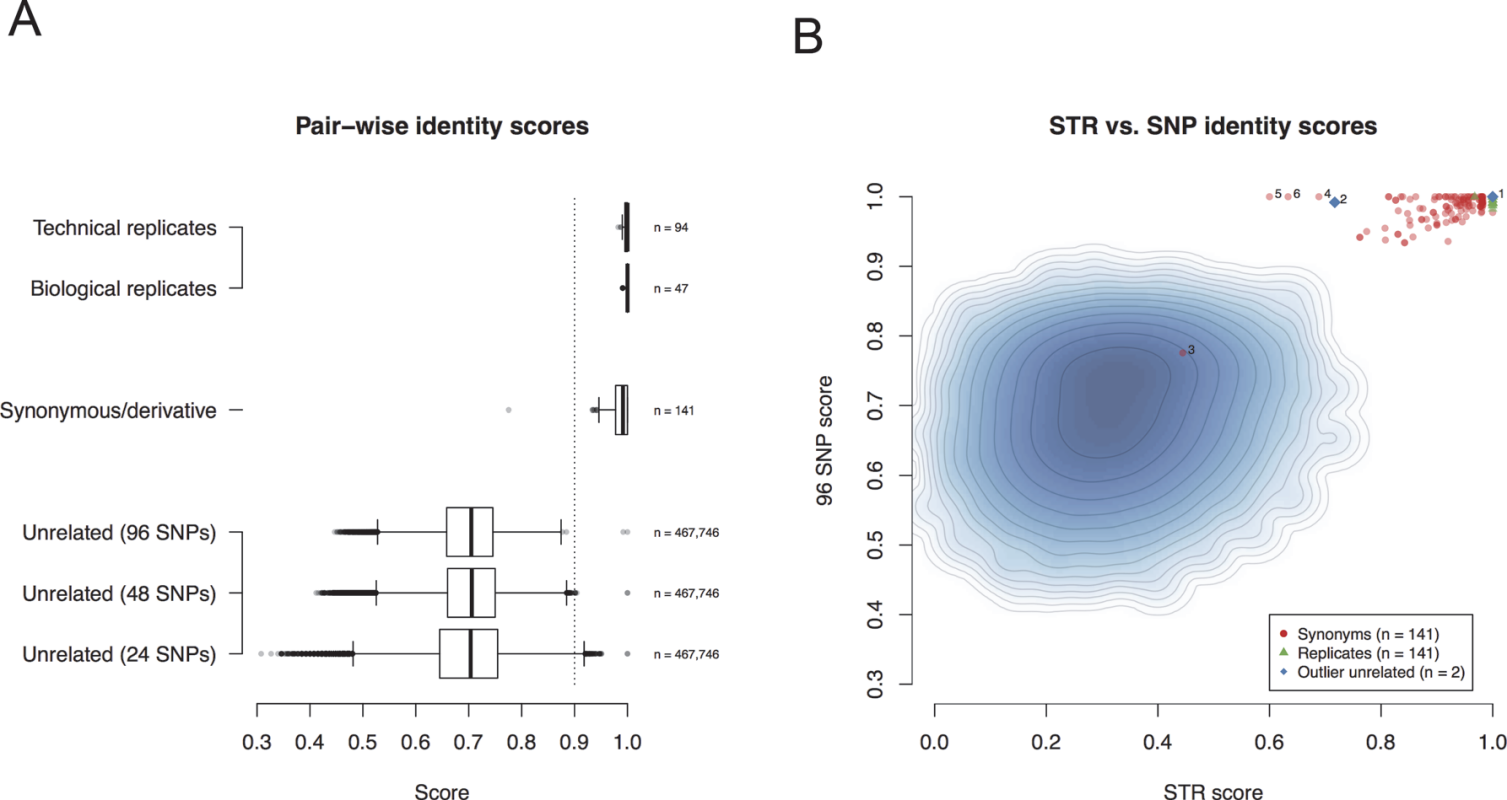
Pairwise identity scores for cell lines using the SNPtrace Panel and STR profiling. (**A**) Results of pairwise analysis of all cell line samples broken out into technical and biological replicates, synonymous samples, and unrelated samples. For unrelated samples, 24- and 48- SNPs were randomly selected for pairwise comparisons and contrasted with the complete 96-SNP set. (**B**) Comparison of pairwise scores from the SNPtrace Panel and STR profiling. Shaded region is joint distribution of SNP and STR-based identity scores for pairwise comparisons of unrelated lines (*n* = 467,746); in addition, two unrelated pairs which generated unexpectedly high identity scores are called out as blue diamonds (“1” and “2”). Red and green points correspond to synonymous and replicate pairs, respectively. Table 1 describes the cell line pairs marked by 1–6 in this figure.

Mean and standard deviation for the pairwise identity scores were computed for all synonymous pairs (excluding two outliers in **Fig. 1B**). The distribution of these scores was seen to be approximately Gaussian, so non-synonymous pairs were scored based on the upper tail probability of a normal distribution with the computed mean and standard deviation.

**Table 1:** List of observed discrepancies between the SNPtrace Panel and STR comparisons.

	Cell line 1	Cell Line 2	Identity score (SNP)	Identity score (STR)	Observation
1	NCI-H2691	NCI-H2803	1.00	1.00	Cell lines are identical, not previously known
2	KM-12	PMF-ko14	0.99	0.72	Cell lines are identical, not previously known
3	COR-L47	COR-L51	0.78	0.44	Cell lines reported as synonymous (Sanger), but have unique profiles
4	HM7	MT-3	1.00	0.69	Identical by SNP, weak identity by STR. Discrepancy likely due to MSI affecting STRs
5	LS-180	MT-3	1.00	0.60	Identical by SNP, weak identity by STR. Discrepancy likely due to MSI affecting STRs
6	LS-174T	MT-3	1.00	0.63	Identical by SNP, weak identity by STR. Discrepancy likely due to MSI affecting STRs

### Illumina SNP Arrays

Illumina HumanOmni2.5_4v1 arrays were used to assay cancer cell lines for genotype, DNA copy and LOH at ~2.2 million SNP positions following methods published previously [24,25].

In brief, we applied a modified version of the PICNIC [26] algorithm to estimate total copy number and allele-specific copy number / LOH. PICNIC was designed to work only with the Affymetrix SNP 6.0 array and thus some elements of the code were modified or replaced. Most significantly, it was necessary to transform the two-dimensional probe intensity data for each SNP so that the normal AA, AB, and BB genotype centroids are separated by one unit in both the horizontal and vertical directions. To achieve this, we first used a Bayesian model to estimate cluster centroids for each SNP, and then applied a smooth, non-linear transform to send each centroid to the appropriate location. Genotypes were obtained using the GenomeStudio (http://bioinformatics.Illumina.com) application (version 2011.1) with the default settings.

Sample sex determination was made using both genotype and copy number data. The AA/AB/BB genotype calls were analyzed to determine the fraction of heterozygous SNPs (AB genotype) on chromosome X. The raw, pre-segmentation copy number ratios (where 1 indicates a copy number equal to the genome average copy number) were analyzed to determine the average value on chromosome Y. Visual inspection of histograms of these two values each indicated bimodal densities. Cutoffs of < = 0.05 for the heterozygous fraction and > 0.5 for chromosome Y copy number ratio were selected to identify male samples (i.e. with only one allele for chromosome X genes and evidence of chromosome Y being present).

The genomic regions (hg19) covering the genes PCDH11Y (chrY:2988462-5587151) and PCDH11X (chrX:88455396-92368901) were excluded from the copy number analysis as they generally show locally increased copy number in putatively male samples. These regions makes up the “PAR3” pseudo-autosomal region [27] of chromosome Y and a potentially cross-hybridizing region on chromosome X. The array does not include SNPs in the PAR1 or PAR2 regions.

Cell line SNP data has been deposited in the European Genome-phenome Archive (EGA). Accession number EGAS00001000610.

### Detection of cross-contamination

To test the sensitivity of detecting cross-contamination, we created cell mixtures, THP-1:SK-MEL-1, AU565:SK-MES-1 and MCF-7: LOX-IMVI at fixed ratios of (100:0, 99:1, 98:2, 95:5, 90:10, 50:50, 5:95, 10:90, 2:98, 1:99, 0:100). Cell counts were determined by ViCELL (Beckman). DNA was extracted from the cell mixtures using the DNeasy Blood & Tissue Kit (Qiagen, cat#69506) following manufacturer’s protocol. DNA concentration was determined by NanoDrop 8000 and normalized to 50ng/ μL for all DNA samples.

Contamination experiments for diploid samples were performed by mixing a human male and female sample. Three pairs were analyzed using DNA supplied by RUCDR Infinite Biologics (Piscataway, NJ). Both male and female samples were diluted to 60ng/μL. Contaminated samples were created by mixing each 60ng/μL sample into the other to create 20%, 15%, 10%, 5% and 1% contamination by volume.

### Spectral Karyotyping (SKY)

Metaphase slides were prepared from cell lines that were cultured, harvested, and fixed with methanol:acetic acid (3:1), according to standard cytogenetic procedures. Seven microliters of denatured SkyPaint probe (Applied Spectral Imaging (ASI), Vista, CA) was added to each denatured metaphase slide, which then was covered by a glass coverslip and incubated overnight in a 37°C humidified chamber. Slide pretreatment and post-hybridization washes were performed according to the standard supplied protocol (ASI) with slight modifications. Image acquisition was performed with a COOL-1300 SpectraCube camera (ASI) mounted on an Olympus BX43 microscope using a SKY optical filter (ASI). For each cell line, a minimum of 10–15 metaphases were analyzed using the HiSKY v6.0 software (ASI).

### Fluorescence *in situ* hybridization (FISH)

FISH probes were prepared from purified BAC clones RP11-91A13 and RP11-71M14 (Yq11.221) and RP11-452D1 (17p13.1/Yq12) (BACPAC Resource Center, bacpac.chori.org) and labeled with Orange-dUTP and Green-dUTP (Abbott Molecular Inc., Des Plaines, IL), respectively, by nick translation. The Yq11.221 FISH probe is specific for chromosomal locus Yq11.221, while the 17p13.1/Yq12 FISH probe hybridizes to chromosomal locus 17p13.1 and also the entire band Yq12, which generates a huge FISH signal on Yq12. Metaphase slides were prepared from 7 of the cell lines previously harvested for SKY analysis [28]. The metaphase slides were pretreated with 2X saline/sodium citrate (SSC) at 37°C for 10 min, 0.005% pepsin/0.01M HCl at 37°C for 4 min, and 1X PBS for 5 min. The slides were then placed in 1% formaldehyde/1X PBS for 10 min at room temperature, washed with 1X PBS for 5 min, and dehydrated in an ethanol series (70%, 85%, and 95%) for 2 min each. Sample slides were denatured in 70% formamide/2X SSC at 73°C for 5 min, washed in a cold ethanol series (70%, 85%, 95%) for 2 min each, and air-dried. FISH probes were denatured at 73°C for 5 min and kept at 37°C for 10–30 min. Eight microliters of probe was applied onto each slide and mounted with a glass coverslip. The slides were hybridized overnight at 37°C, washed with 2X SSC at 73°C for 2 min, and rinsed briefly in distilled water. Slides were air-dried, counterstained with VECTASHIELD mounting medium with 4'-6-diamidino-2-phenylindole (DAPI) (Vector Laboratories Inc., Burlingame, CA), and coverslips were applied. Image acquisition was performed at either 600x or 1000x system magnification with a COOL-1300 SpectraCube camera (ASI) mounted on an Olympus BX43 microscope. Images were analyzed using FISHView v7.2.6 software (ASI), and at least 20 metaphases were scored for each cell line, with the exception of NCI-H441, where 100 cells were scored due to low level presence of Chr Y.

Reverse DAPI images were acquired through the FISHView analysis software by inverting the DAPI to a black and white image, which allows for visualization of chromosome centromeres and bands similar to G-banding.

## Results

### SNPtrace Panel profiles of 907 human cell lines

The SNPtrace Panel simultaneously assesses 96 SNPs, consisting of 90 autosomal and 6 allosomal SNP assays, and interrogates 96 samples in a single run. In total, including biological and technical replicates, we analyzed 968 samples using the SNPtrace Panel obtaining an autosomal call rate of 99.34%, with just 574 out of 87,120 autosomal SNP “No Calls” (**S1 Table**). Of the samples run, there were 907 individual cell lines of which 820 represent unique individuals. The remaining samples consist of cell lines known to be either derived from the same patient (termed “synonymous”), misidentified or derivatives of parental lines. To evaluate the accuracy with which the SNPtrace Panel can discriminate between related and unrelated cell lines, pairwise comparisons were performed for all samples (see Materials and Methods). Biological and technical replicates performed as expected with a high degree of concordance. All replicate pairs produced *p*-values below 6.0 x 10^−7^ when tested against the identity score distribution observed for unrelated pairs of lines. With one exception (discussed below), synonymous/derivative pairs of cell lines (those derived from the same patient or from a common parental line) were also highly similar (all *p* < 2.9 x 10^−5^) and thus could be identified by the SNPtrace Panel (**Fig. 1A**). For the samples under consideration, a cutoff of ≥ 90% identity was sufficient to discriminate between the majority of related and unrelated samples (**Fig. 1A**). To assess the discriminatory power of 96 SNPs compared to smaller numbers of SNPs, we randomly selected 24 and 48 autosomal SNPs from the SNPtrace Panel, performed pairwise comparisons for each group and compared the distributions of these identity scores with the full panel. As expected, there was a decrease in the variability of identity scores for unrelated pairs as more SNPs were used, indicating an increased discriminatory power [18,29–31] (**Fig. 1A**). Specifically, although the mean identity score for unrelated pairs changed negligibly when SNP counts were reduced to 48 or 24, standard deviation (SD) increased significantly (by 11% for 48 SNPs, from 0.058 to 0.065; and by 34% for 24 SNPs, from 0.058 to 0.078).

STRs are the ANSI standard for cell line authentication [12,13,32]. We generated STR profiles for all cell lines analyzed by the SNPtrace Panel and contrasted pairwise comparisons of the STR profiles with those of the SNP profiles to assess the relative discriminatory power of both assays. This comparison showed that the SNPtrace Panel is comparable to STR profiling at discriminating between related and unrelated samples, and that biological and technical replicates scored equally well in both assays (**Fig. 1B**). This analysis identified some unexpected findings outlined in **Table 1**, including identification of two previously unknown pairs of identical cell lines (NCI-H2691/NCI-H2803 and KM-12/PMFko14), and identification of unique profiles for a pair of lines previously thought to be synonymous (COR-L47/CORL51, “3” in **Fig. 1B**). We also found high concordance, as measured by the SNPtrace Panel, between four lines reported as synonymous (HM7, LS-174T, LS-180, and MT-3, with comparisons to MT-3 indicated as "4", "6" and "5," respectively, in **Fig. 1B**) [33,34], which had borderline/low concordance by STR. These findings were confirmed by the Illumina SNP arrays. These lines are known to have microsatellite instability (MSI) which may affect STR results more than SNPs [35–37]. We also identified one sample handling error (EFM-192B) which was corrected and thus does not appear in Fig. 1 or supplementary material. Our findings highlight the need for continuous cell line profiling for quality control.

### Cell line sex determination

Unlike other reported SNP profiling panels [29–31], the SNPtrace Panel includes six SNPs to determine the sex of the sample (three each for X and Y). Historically, STRs have a high rate of calling male samples female, presumably due to loss of the amelogenin locus located on chromosome (Chr) Yp11.2 [38–40]. We compared sex calls made by (i) STR, (ii) Illumina SNP arrays (*Klijn et al*. *In press*, *Nat Biotech*) and (iii) the SNPtrace Panel with annotated sex calls. All three assays performed similarly, identifying 40–45% of males as females but only 2–7% of annotated female cell lines as male with a high degree of concordance between assays (Table 2). The high level of consistency between the three platforms suggested the discrepancy may not be due to a technical issue and prompted us to compare with published karyotypes for 19 cell lines analyzed by Spectral Karyotyping (SKY) [24]. Table 3 compares the sex calls for the SNP- and STR-based assays with allosome calls from the SKY karyotyping for these cell lines. Out of 19 cell lines, 7 were annotated as male but called female (using the Illumina SNP data), and all 7 samples had no evidence of any Chr Y material by SKY (with the exception of a small portion of Yq found only in NCI-H522). Interestingly, there was no evidence of a Chr Y by SKY in NCI-H650, which was called male by Illumina SNP array (based on copy number of Chr Y) but female by STR and the SNPtrace Panel. In addition, NCI-H441 lacked Chr Y by SKY but was called male by both STR and SNP assays. In light of these inconsistencies, and since the karyotypes indicated a high degree of aneuploidy and complex chromosomal rearrangements, we hypothesized that fragments of Chr Y may be present in these aberrant genomes which were missed by SKY. To test this, we performed Chr Y FISH to detect Chr Y fragments integrated into other chromosomes (**S2 and S3 Tables**). No evidence of Chr Yq was found in 4 of the 7 male lines tested which typed as female (NCI-H23, NCI-H322T, NCI-H838 and NCI-H1299). In NCI-H522, we detected 1 copy of Chr Yq12 (der(15)t(Y;15)(q11.22?2;p11.1)) by SKY and FISH, suggesting that Chr Y was present but what remains of this portion of Chr Y apparently is not detectable by the other methods. Although SKY indicated no presence of Chr Y in NCI-H441, FISH and reverse DAPI banding detected a normal Chr Y in 18% of cells, explaining why the PCR-based STR and the SNPtrace Panel assays detect Chr Y. On closer inspection, the Illumina SNP arrays did, in fact, detect a low level of Chr Y, but this was missed by the cut-offs we used to identify males (**S1 Fig.**). The positive control, NCI-H460, showed 1–2 copies of Chr Y per cell by SKY, FISH and reverse DAPI banding.

**Table 2:** Comparison of sex calls derived from STR profiling, the SNPtrace Panel and Illumina SNP arrays with annotated sex calls for all cell lines.

	Analysis Calls					
	STR		SNPtrace		Illumina Array	
Annotated	Male	Female	Male	Female	Male	Female
Male	220 (55%)	180 (45%)	219 (60%)	144 (40%)	194 (60%)	131 (40%)
Female	8 (2%)	382 (98%)	15 (4%)	327 (96%)	23 (7%)	313 (93%)
Total	790		705		661	

**Table 3:** Comparison of sex calls derived from STR profiling, the SNPtrace Panel, Illumina SNP arrays and SKY.

Call (based on SNP array)	Cell Line Name	CLID	Sex calls				
			Annotated	STR	SNPTrace	illumina	SKY Karyotype
Female typed as Female	LXFL529	130847	Female	Female	Female	Female	XX
	NCI-H1993	129701	Female	Female	Female	Female	XXXXXX
	NCI-H2009	131971	Female	Female	Male	Female	X
	NCI-H2073	129695	Female	Female	No Call	Female	XXX
	NCI-H2122	130148	Female	Female	Female	Female	XX
	NCI-H292	129184	Female	Female	No Call	Female	XX
Male typed as Female	NCI-H1155	131718	Male	Female	No Call	Female	XX
	NCI-H1299	129461	Male	Female	No Call	Female	XXXX
	NCI-H23	129190	Male	Female	Female	Female	XX
	NCI-H322T	585717	Male	Female	Female	Female	XX
	NCI-H441	130150	Male	Male	Male	Female	XX
	NCI-H522	129185	Male	Female	Female	Female	XX
	NCI-H838	129191	Male	Female	Female	Female	X
Male typed as Male	A549	130497	Male	Male	Male	Male	XXY
	NCI-H1703	130536	Male	Male	Male	Male	XY
	NCI-H226	129544	Male	Male	Male	Male	XYY
	NCI-H358	129179	Male	Male	Male	Male	XXY
	NCI-H460	129455	Male	Male	Male	Male	XXYY
	NCI-H650	129187	Male	Female	Female	Male	XXX

These data suggest that many of the cancer cell lines derived from male patients which profiled as female may have lost the entire Y chromosome, not just the amelogenin locus, or may have dramatically rearranged genomes with fragments of Chr Y translocated to other chromosomes.

### Cross contamination

**Fig 2:**
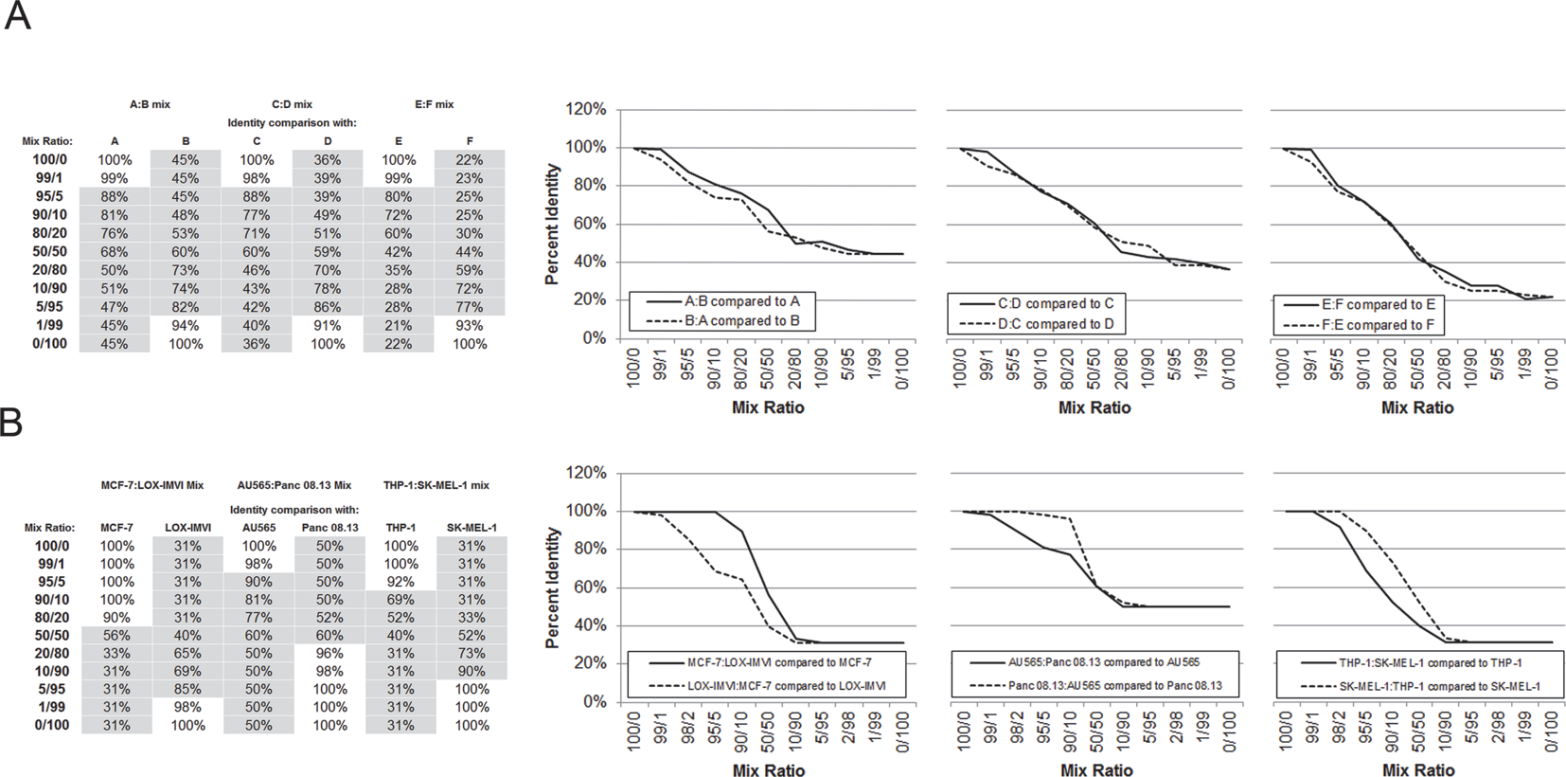
Detection of cell line cross-contamination by the SNPtrace Panel. DNA from two different normal human samples (**A**) and tumor cell lines (**B**) were mixed as indicated at the specified ratios, and analyzed by the SNPtrace Panel. Tables A and B include the calculated percent identity for each sample pair, and percent identities highlighted in grey indicate a non-identical call (<90%). Plots to the right of each table show the overlay of percent identities for reciprocal mixes.

Cell line cross-contamination is an acknowledged and major problem in biomedical sciences [10], and STRs have been reported to reliably detect a contaminant at 5–10% [41]. To test the sensitivity of the SNPtrace Panel to detect cell contaminants, we performed cell mixing experiments (see Materials and Methods). Initially, these were performed on normal (diploid) human DNA, and the percent identity for SNP calls was calculated between the mixes and both the parental samples. Using an identity score cutoff of 90% to call samples as identical, the SNPtrace Panel could reliably detect 5% contamination in three independent mixes. For diploid cells, identity was not affected by the sample chosen, as reciprocal mixes resulted in similar identity scores with each individual sample (**Fig. 2A**). We performed the same test on three pairs of tumor cell lines and found that sensitivity ranged from 2%-10% depending on the ratio of the samples, and equivalent reciprocal mixes were often not detected with the same degree of sensitivity. For example, MCF-7 was only detected at 50% contamination (a 50:50 mix ratio in the MCF-7:LOX-IMVI mixture), whereas just 2% contamination of LOX-IMVI was detected (a 98:2 mix ratio in the MCF-7:LOX-IMVI mixture) (**Fig. 2B**). Similar results were observed using STR analysis of these samples (data not shown). Our best interpretation of this data is that the degree of aneuploidy and copy number at any given locus may influence the sensitivity of the assay and thus make contamination by a high copy number cell line easier to detect than contamination by a line with fewer copies.

## Discussion

DNA-based authentication is required to validate the results of any research using human biosamples. However, there is a remarkable lack of attention to the problem of cell line misidentification by researchers [42]. Some journals and grant-giving organizations are beginning to request cell line authentication; however, the problem is not completely solved, as most do not require a definitive confirmation of authentication and in many cases papers are published without any assurance [43]. There are excellent resources for learning and implementing good cell line practices [44], and these can be broken down into a few basic principles: understanding the model, quality assurance, documentation/tracking of methods, reducing exposure to hazards, ethical compliance and education to ensure highest quality of work and safety [45]. Many institutes enforce training and safety aspects, but do not enforce quality assurance of cell lines (and reagents) used in biomedical research which is arguably the most important aspect to ensure reliable and reproducible data.

STR analysis is the ANSI standard for profiling cell lines. A wealth of cell line STR profiles are available for comparison (although there is no comprehensive, centralized reference database), and a growing number of organizations offer fee-for-service STR profiling (outsourcing STR analyses can range from $100–295). As an alternative, SNP-based profiling has been suggested as a cost-effective, simple alternative [29–31]. Here we assessed the performance of a new commercial SNP profiling assay, the SNPtrace Panel, and compared it directly with STR profiling of a panel of human cell lines. We found that the SNPtrace Panel showed equivalent performance to STR profiling, that 96 SNPs outperformed 48 and 24 SNPs, and that a threshold of 90% SNP identity was able to confidently discriminate between related and unrelated samples. In samples with microsatellite instability, SNP profiling appeared to outperform STR profiling in identifying related samples [37]. The system is high-throughput (96-samples per run), and commercial reagent costs are approximately $6 per sample, compared to $15–30 for STR analysis. Hands-on sample handling for 96-samples is 50 min for SNPs, compared to 3 h for STR; machine run time for 96-samples is 4 h 35 min for SNPs, compared to 18 h for STR. In our experience the time needed for data analysis is equivalent for both platforms.

Similar to others, we found that SNP-based profiling can detect intra-species cross-contamination as low as 2% with a range of 2–10% [30], which compares favorably with STR. However, we observed that the sensitivity is dependent on the samples and the ratio of mixing for cancer cell lines for both methods. We believe this is due to aneuploidy in cancer cell lines, but this remains to be formally tested. Therefore we advise frequent profiling of cell line stocks to catch outgrowth of low-level contaminants over time.

There is a high rate of calling male samples female by STR [38,39]. When we compared sex calls from three different platforms, we noted that an astounding 40–45% of cell lines annotated as male were called female by all three methods we considered. Since these three methods are quite distinct, it seemed unlikely that loss of the amelogenin locus alone was responsible, and we tentatively discounted human error since it would be expected that a similar number of samples annotated as female would be misidentified as male, which was not the case (Table 2). We have also sex typed a number of Coriell (diploid) cell lines without experiencing miscalls (data not shown). Some level of misdetection is inherent with all three methods we used. For example, the copy number algorithm we used can detect a mixture of normal and cancer cells, but it currently cannot detect sub-populations of cancer cells with copy number differences within specific chromosomes. In the case of NCI-H441, the allele ratios for chromosome Y SNPs were non-zero, but were closer to the expected values for zero copies than to those for one copy, resulting in a false negative call (**S1 Fig.**). Such discrepancies required cytogenetic analysis which clearly showed that loss of most or all of Chr Y was occurring. A more detailed cytogenetic analysis of cell lines and the tissue of origin (if available) is required to formally prove if this is a common genomic feature for cell lines in culture, or represents a worryingly high level of poorly annotated cell lines. Our data suggest that Chr Y loss is due to genomic instability as residual Chr Y fragments were found in some samples. While it is clear that it is challenging to accurately determine the sex of a genomically unstable cell line, the autosomal calls are definitive for identifying the origin of a cell line.

In summary, we demonstrated that the SNPtrace Panel is a DNA profiling technology which could be used alone or in conjunction with established cell line authentication methods for labs to continually monitor cell line identity. We have provided a resource of 907 SNP profiles for future comparisons and have demonstrated that SNPs provide a fast, reliable, accurate and cost-effective method for assessing cell line or human biosample identity and intra-human cross-contamination.

## Supporting Information

S1 Fig Theta values [26] for chromosomes X and Y from three cell lines. Theta, a measure much like the B-allele frequency, gives a measure of the relative abundance of the so-called "A" and "B" alleles. A value of ~0.5 indicates heterozygosity. (**A**) A549 has normal sex chromosomes for a male. The theta values indicate the absence of heterozygous alleles on chromosome X, with the exception of the PAR1 and PAR3 regions (PAR2 is covered by only one SNP on the array). On chromosome Y, theta indicates the presence of one copy of chromosome Y. (**B**) NCI-H1045 has normal female sex chromosomes. Theta indicates clear evidence for heterozygous alleles on chromosome X and the signal on chromosome Y is characteristic of homozygous deletion or the lack of chromosome Y. (**C**) NCI-H441 shows no evidence of heterozygous alleles on chromosome X. The signal on chromosome Y is intermediate between that of (**A**) and (**B**), suggesting a mix of cells with one chromosome X and one chromosome Y and cells with just one chromosome X.
(TIF)

S1 Table Cell lines, annotations, SNPtrace Panel results and STR profiles for samples used in this study. Index: simple numerical identifier; Sample Count: Number of times the sample was run; Replicate type: TR = Technical Replicate, BR = Biological Replicate,— = unique sample; Cell line name: name of cell line; CLID: unique identifier used within Genentech (this identifier can be used to compare with other published data from Genentech); Species: species from which the cell line was derived; Sex: annotated sex of cell line; Primary Tissue: the tissue from which the cell line originated; Tissue Diagnosis: Type of disease the cell line was derived from; Synonymous/derivatives: known synonymous or derivative cell lines; Number of synonyms in dataset; the count of any synonymous/derivative cell lines present in the data set related to this sample; # No call; the number of autosomal no calls for the SNPtrace Panel results. All headers for the SNPtrace Panel and STR data indicate the SNP or STR identifiers.
(XLSX)

S2 Table Chr X / Chr Y karyotypes for 19 cell lines. Table describing the detailed karyotype of 19 cell lines and the inferred sex type.
(XLSX)

S3 Table Chr Y FISH results. Table describing the detailed results of Chr Y FISH for 7 cell lines.
(XLSX)

S4 Table dbSNP (“rs”) identifiers and chromosomal locations of the SNPtrace Panel SNPs. (XLSX)

S5 Table SNPtype thermal cycling and pre-amp conditions. (XLSX)
