# Supplementary figures and images for: Human Biosample Authentication Using the High-Throughput, Cost-Effective SNPtraceTM System
Source: PLoS One. 2015 Feb 25;10(2):e0116218. doi: 10.1371/journal.pone.0116218 (PMC4340925; doi:10.1371/journal.pone.0116218)

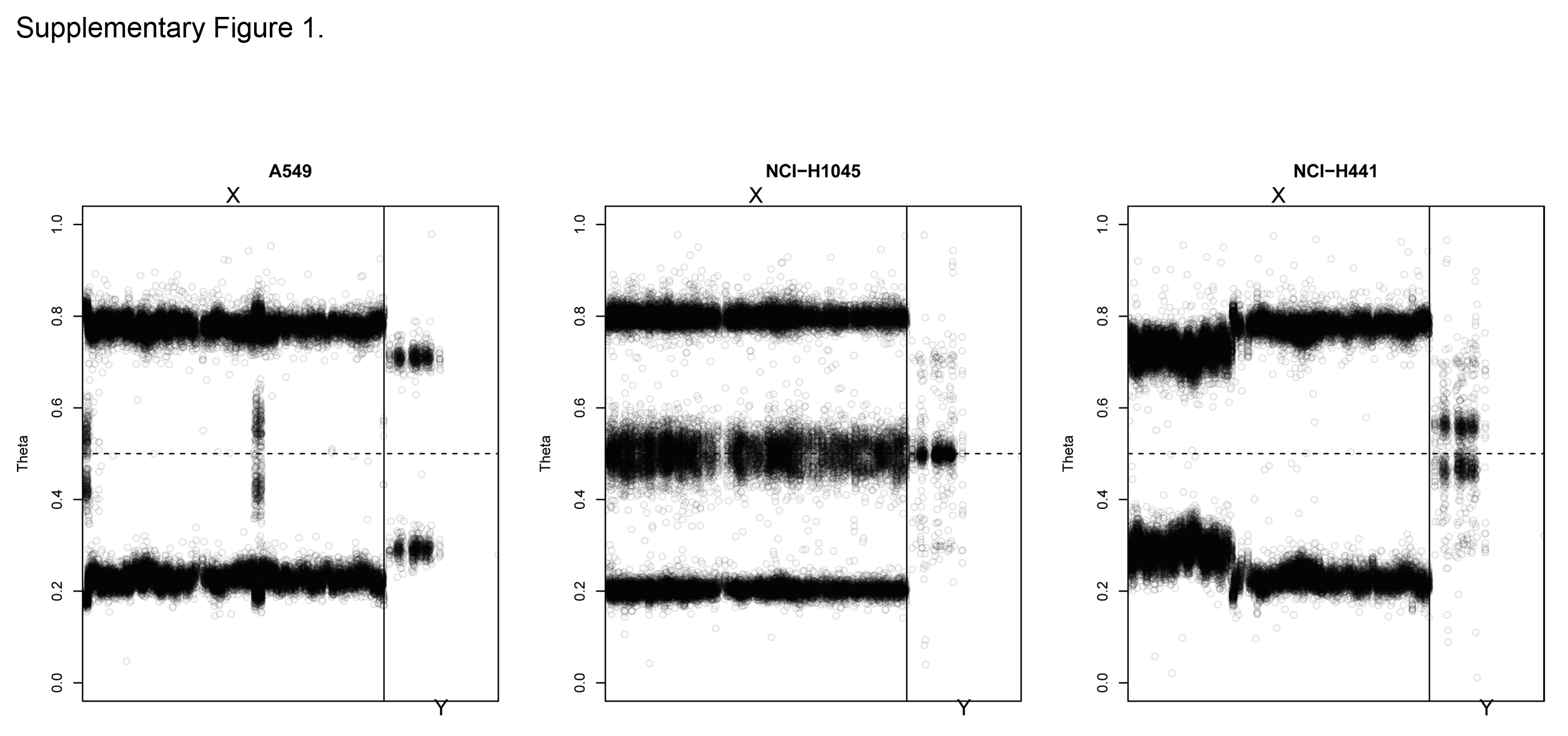

Supplement: S1 Fig — Theta, a measure much like the B-allele frequency, gives a measure of the relative abundance of the so-called "A" and "B" alleles. A value of ~0.5 indicates heterozygosity. (A) A549 has normal sex chromosomes for a male. The theta values indicate the absence of heterozygous alleles on chromosome X, with the exception of the PAR1 and PAR3 regions (PAR2 is covered by only one SNP on the array). On chromosome Y, theta indicates the presence of one copy of chromosome Y. (B) NCI-H1045 has normal female sex chromosomes. Theta indicates clear evidence for heterozygous alleles on chromosome X and the signal on chromosome Y is characteristic of homozygous deletion or the lack of chromosome Y. (C) NCI-H441 shows no evidence of heterozygous alleles on chromosome X. The signal on chromosome Y is intermediate between that of (A) and (B), suggesting a mix of cells with one chromosome X and one chromosome Y and cells with just one chromosome X. (TIF) [file pone.0116218.s001.tif]
